# Working memory capacity moderates the effect of hearing aid experience on phonological processing performance

**DOI:** 10.3389/fnins.2025.1519934

**Published:** 2025-02-18

**Authors:** Ruijing Ning, Emil Holmer, Henrik Danielsson, Carine Signoret, Andreea Micula, Jerker Rönnberg

**Affiliations:** ^1^Disability Research Division, Department of Behavioural Sciences and Learning, Linköping University, Linköping, Sweden; ^2^Department of Public Health, University of Copenhagen, Copenhagen, Denmark; ^3^Linnaeus Centre HEAD, Department of Behavioural Sciences and Learning, Linköping University, Linköping, Sweden

**Keywords:** rhyme judgment, hearing impaired, language skill, hearing aid use, cognitive performance

## Abstract

**Purpose:**

Individuals with acquired hearing impairment often experience declines in phonological processing abilities, a phenomenon thought to be mediated by working memory capacity (WMC). However, the role of hearing aid use in this context remains underexplored. Therefore, in the current study, we aimed (1) to tease apart the effect of hearing impairment and hearing aid use on phonological processing performance, and (2) to investigate the effect of hearing aid use on phonological processing in more detail, while considering the involvement of WMC.

**Method:**

Using mixed effect models, we investigated rhyme judgment performance and its reliance on WMC among three groups of participants: a group of hearing aid users (*n* = 202), a group of hearing-impaired individuals without hearing aid (*n* = 54), and a group of normal hearing controls (*n* = 201). We also examined how years of hearing aid use was associated with rhyme judgment performance and its reliance on WMC in hearing aid users.

**Results:**

We found that hearing impairment was associated with increased dependence of rhyme judgment performance on WMC regardless of the use of hearing aids. Furthermore, hearing aid use was overall positively associated with rhyme judgment performance, with this relationship influenced by variations in WMC.

**Conclusion:**

WMC influences the effectiveness of hearing aids for phonological processing. This role may result from working memory’s involvement in the processing of output signals from hearing aids, as well as in the strategies hearing aid users implement to deal with phonological processing tasks.

## Introduction

Hearing loss causes disadvantages in many aspects of life beyond the direct impact on auditory perception. A plethora of recent studies pointed to the association between age-related hearing loss and cognitive decline (Bucholc et al., 2022; Lin et al., 2013, 2023; Livingston et al., 2017; Maharani et al., 2019; Rönnberg et al., 2011, 2014). Hearing loss appears as a risk factor for cognitive impairment such as dementia (Lin et al., 2013; Livingston et al., 2017), mild cognitive impairment (MCI) (Bucholc et al., 2022), and memory-related problems (Maharani et al., 2019), as well as a negative effects on non-auditory encoding conditions in episodic verbal free recall tasks (Rönnberg et al., 2011, 2014). This implies that the negative effect rests with memory system, independent of encoding modality, and that especially long-term memory systems are affected (Rönnberg et al., 2011).

Hearing aid use is a prevalent hearing rehabilitative strategy to reduce the adverse effects of hearing impairment. Growing evidence from observational studies shows that hearing aid use is associated with better cognition and reduced risk of dementia (Amieva et al., 2015; Dawes et al., 2015; Lin et al., 2023). Research on the effect of hearing aid use on language production and comprehension is more limited. While several studies have examined the effect of short-term (under 1 year) hearing aid use on language tests, they found no significant improvement (for a review, see Sanders et al., 2021). However, few studies have examined the effect of hearing aid use on the underlying cognitive abilities important for language production and comprehension (but see, e.g., Ng and Rönnberg, 2020). This study aimed to examine the relationship between hearing loss, hearing aid use, and phonological processing—a cognitive process involved in language production and recognition—with a focus on the moderating effect of working memory (cf. Homman et al., 2023; Rönnberg et al., 2013, 2022).

The processing of phonological information—using the mental representation of the spoken sounds and combinations of sounds of the words stored in long-term memory—is involved in spoken and written language production and perception (Goswami, 2012; Rönnberg et al., 2016). The rhyme judgment task, where participants judge whether two written items rhyme or not, is often used to examine phonological processing. When the two items are presented, the participants need to access, store, and manipulate the phonological representations to perform the task (Classon et al., 2013a; Oakhill and Kyle, 2000). Presented visually as printed words or pictures, the task has the advantage of examining phonological processing without auditory or verbal output, making it a useful tool in populations with hearing or speech impediments.

Individuals with hearing impairment have been shown to perform worse in rhyme judgment tasks than individuals with normal hearing (Andersson and Lyxell, 1999; Andersson, 2002; Classon et al., 2013a; Lazard and Giraud, 2017; Lyxell et al., 1994, 1998; MacSweeney et al., 2013). Congenitally deaf individuals (MacSweeney et al., 2013) and post-lingually deafened individuals (Lazard and Giraud, 2017; Lyxell et al., 1994, 1998) showed a lower accuracy in rhyme judgment task compared to individuals with normal hearing. Individuals with acquired moderate-to-severe hearing impairment also showed lower accuracy (Andersson, 2002; Classon et al., 2013a) and longer reaction times (Andersson, 2002) in rhyme judgment task compared to individuals with normal hearing. Several studies also found that the decrease in accuracy correlated with the duration of hearing impairment (Classon et al., 2013a; Lyxell et al., 1994, 1998). These findings imply that hearing impairment has a detrimental effect on phonological processing ability, probably caused by auditory distortion and auditory deprivation (for a review, see Lyxell et al., 2003).

Notably, in individuals with acquired hearing loss, working memory seemed to play a more important role in rhyme judgment task than in individuals with normal hearing (Andersson, 2002; Classon et al., 2013a). Working memory is responsible for temporarily holding and processing information during ongoing cognitive processes (Baddeley, 1992). Classon et al. (2013a) found that rhyme judgment accuracy was independent of working memory capacity (WMC) measured with the reading span test (RST, Rönnberg et al., 1989) in normal-hearing participants. However, rhyme judgment accuracy was predicted by WMC in participants with moderate-to-severe hearing loss. Similarly, rhyme judgment accuracy correlated with verbal WMC measured with the word span test (Baddeley et al., 1975) in participants with hearing impairment but not those with normal hearing (Andersson, 2002). A closer look at individuals with hearing impairment found that individual differences in rhyme judgment reaction times were also explained by WMC measured with the Reading Span Task (RST) (Rudner et al., 2019). These findings demonstrate that, when performing the rhyme judgment task, individuals with hearing impairment compensate for the deterioration of phonological processing through strategies that require working memory. Findings from an electroencephalographic study also suggests that individuals with hearing impairment relied more on compensatory top-down strategy such as articulatory recoding and grapheme-to-phoneme conversion procedures to enhance the precision of phonological/orthographical information held in working memory (Classon et al., 2013b).

Nevertheless, in the studies reporting a correlation between rhyme judgment performance and WMC in individuals with hearing impairment, it is worth noting that the hearing-impaired participants were all experienced hearing aid users (Andersson, 2002; Classon et al., 2013a, 2013b; Rudner et al., 2019). This brought a complicating factor into the findings: it was possible that hearing aid use was associated with the differences in performance, especially the enhanced involvement of WMC in rhyme judgment performance. This would put into question the argument that it was hearing impairment alone that led to the enhanced involvement of WMC.

In fact, very little attention has been paid to the effect of hearing aid use on phonological processing. Rudner et al. (2019) reported that hearing aid experience did not account for a significant amount of variance in the visual rhyme judgment task beyond lexical access speed and WMC. However, this study did not consider the possible interaction between hearing aid experience and WMC. It was possible that the moderating effect of WMC contributed to this null outcome because the effect of hearing aid use may change for individuals with different WMC, and hearing aid use may change how task performance relies on WMC (Lunner et al., 2009). A moderating effect of WMC has been found when investigating the effect of hearing aid use on speech perception in noise, showing that even after long periods of hearing aid use some conditions never seem to reach complete independence of WMC (Ng and Rönnberg, 2020). This may in turn imply that some conditions which are sufficiently dynamic in their matching to stored long-term memory representations are too variable to create entirely reliable prototypical representations that capture dynamic stimuli in the long-term (Rönnberg et al., 2022).

Therefore, as highly relevant to the current literature, we aimed to (1) distinguish the effect of hearing impairment on phonological processing from the influence of hearing aid use, and to (2) investigate the relationship between hearing aid use and phonological processing in more detail, while considering the involvement of WMC. Taking advantage of available data from the n200 project (Rönnberg et al., 2016), we compared the rhyme judgment accuracy and speed among three groups of participants: a group of hearing aid users, a group of hearing-impaired individuals without hearing aid, and a group of normal hearing controls. We predicted that hearing impairment, irrespective of hearing aid use, would be associated with a greater reliance on WMC for phonological processing. Specifically, we expected the association between WMC and rhyme judgment performance to be stronger in both groups with hearing impairment (hearing aid users and non-users) compared to the normal-hearing control group. This prediction is based on prior findings suggesting that individuals with hearing impairment implement compensatory cognitive strategies that involve WMC (Andersson, 2002; Classon et al., 2013a). We further explored whether hearing aid use interacts with WMC in predicting rhyme judgment performance among hearing aid users. This interaction would reflect the complex relationship between cognitive resources and the processing of auditory signals from hearing aids.

## Method

### Participants

The data presented in this study is part of the n200 database (Rönnberg et al., 2016). In this database, a total of 504 individuals is included, of which 215 were hearing-aid users with bilateral, symmetrical sensorineural hearing loss in the range mild-to-severe (the HA group, 91 females, mean age = 60.76), 71 had bilateral, symmetrical sensorineural hearing loss in the range mild-to-severe but had not used hearing aids (the noHA group, 25 females, mean age = 69.09), and 218 were normal hearing non-hearing aid users (the NH group, 107 females, mean age = 61.53). The HA group was recruited from the patient population at the Linköping University Hospital, and the NH group was recruited via mail based on information from SCB (Statistics Sweden). Some participants recruited as the NH group turned out to have hearing loss, and were thus identified as the noHA group. All participants gave informed consent and were native Swedish speakers with normal or corrected-to-normal vision, and no reports of neurological diagnoses. The study was approved by the Linköping regional ethical review board (Dnr: 55–09 T122-09).

### Procedure

As a part of the n200 project, participants underwent a battery of tests regarding hearing, cognitive, and executive functions distributed in three testing sessions. Tasks relevant to the current study were carried out in the first and the second session (for details, see Rönnberg et al., 2016).

### Tasks

#### Rhyme judgment task

The visual rhyme judgment task is commonly used to test phonological skills, especially in individuals with hearing impairment (e.g., Booth et al., 2004; Classon et al., 2013a; Classon et al., 2013b; Lazard et al., 2010; Pillay et al., 2014; Rugg and Barrett, 1987). In each trial of the rhyme judgment task, the participant was presented with two printed words on a computer screen, and asked to indicate whether or not they rhymed with each other by pressing buttons (the green button on the right to indicate ‘rhyme’ and the red button on the left to indicate ‘not rhyme’). There were 32 word pairs in total, of which 16 were rhyming (R+) pairs and 16 were non-rhyming (R–) pairs. Within the set of both rhyming and non-rhyming pairs, there were an equal number of orthographically similar (O+) and orthographically dissimilar (O–) pairs. Therefore, word pairs in the following four categories were included:

1. R + O+. Words that rhyme and are orthographically similar, e.g., FRITT/VITT (i.e., free/white).
2. R + O–. Words that rhyme but are not orthographically similar, e.g., KURS/DUSCH (i.e., course/shower in Swedish pronounced [kuʃ/duʃ]).
3. R–O+. Words that do not rhyme but are orthographically similar, e.g., TAGG/TUGG (i.e., thorn/chew).
4. R–O–. Words that neither rhyme nor are orthographically similar, e.g., HÄST/TORN (i.e., horse/tower).

We then grouped the four categories into two types to capture the effect of difficulty on task performance: the mismatching (R + O– and R–O+) trials, in which the orthographic information was a wrong indicator of rhyming, were expected to be harder to solve than the matching (R + O+ and R–O–) trials, in which the orthographic information was a correct indicator of rhyming (Andersson, 2002; Classon et al., 2013a; Rudner et al., 2019). The accuracy and response time for each trial were recorded.

#### WMC measures

With the intention to extract a general WMC factor, we included three measures of WMC: reading span, semantic word pair, and visual spatial working memory. These are all visually based complex span tests which were used to measure the ability to process and to store information simultaneously (see Rönnberg et al., 2016 for details).

##### Reading span

A Swedish version of Daneman and Carpenter (1980) Reading Span test was used to assess WMC (Rönnberg et al., 1989). The test consists of lists of sentences, each of which comprises 2–5 sentences. The sentences were presented in a word-by-word fashion on a computer screen at a rate of one word per 800 ms. After each sentence participants were asked to report whether the sentence was absurd or sensible. After being presented with a list of sentences, participants were asked to report either the first or the last words of each sentence in the list, in their correct serial presentation order. A total of 28 sentences were presented. The test was scored by the total number of items correctly recalled irrespective of recall order.

##### Semantic word pair

A Semantic word pair Test (see Maki, 2007, for materials) is a working memory test that does not include syntactic elements. The test consists of lists of word-pairs, each of which comprises 2–5 word-pairs. The word pairs were presented in a word-pair by word-pair fashion on a computer screen. For each word-pair the participants were asked to identify which word in a word-pair was a living object and give response by pressing buttons. After being presented with a list of word-pairs, the participants were asked to orally recall the word on either the left- or right-hand side in the pair, in their correct serial presentation order. A total of 42 word-pairs were presented. The test was scored by the total number of words correctly recalled irrespective of recall order.

##### VSWM

A Visuo-Spatial Working Memory test is a working memory test that does not include verbal elements. The test consists of a series of trials, each of which comprises 2–5 comparisons. In each trial, a 5 × 5 grid was presented on the screen, and a pair of ellipses, of either identical or different shapes, were presented in one square in the grid. In each trial the participants were asked to judge verbally whether the ellipses were identical or not. After being presented with a series of trials, the participants were asked to note on a sheet of paper with an empty grid the squares in which the ellipses had been presented, in their correct serial presentation order. A total of 42 trials were presented. The test was scored by the total number of squares (where the ellipses had been presented) correctly recalled irrespective of recall order.

### Data analysis

To address the two research questions of this study, we conducted two sets of analysis, one focusing on the group difference among the three groups (group analysis), and another focusing on hearing aid experience in the HA group (hearing-aid experience analysis).

### Missing data and imputation

Participants that did not complete the rhyme judgment test (*n* = 27) or all of the three working memory tests (*n* = 8), or did not provide age data (*n* = 2) were excluded from further analysis. Single trials with reaction time shorter than 100 ms were also removed, for such quick reaction times in the population being tested usually result from mistakes in button pressing. As a result, 8 trials by 7 participants were removed. As a result, a total of 467 participants from the three groups were included in the group analysis (the HA group: *n* = 212, of which 91 were females; the noHA group: *n* = 55, of which 25 were females; the NH group: *n* = 202, of which 107 were females). Table 1 shows the mean age, hearing level, weighted WMC score, and rhyme judgment performance for each group. Of the 212 participants in the HA group that were included in the group analysis, an additional 16 participants were excluded from the hearing-aid experience analysis because of missing data on hearing-aid experience or PTA, resulting in 196 participants for the hearing-aid experience analysis.

**Table 1:** The descriptive statistics of age, hearing level, weighted WMC score, and rhyme judgment performance in each group.

	Total	HA	noHA	NH
	(*n* = 467)	(*n* = 212)	(*n* = 54)	(*n* = 201)
Age(year)				
Mean (SD)	62.01 (± 8.83)	60.70 (± 8.81)	68.96 (± 7.39)	61.53 (± 8.36)
Better-ear PTA (dB HL)				
Mean (SD)	24.89 (± 15.57)	37.57 (± 11.14)	28.66 (± 6.30)	10.40 (± 6.22)
Missing	5 (1.1%)	2 (0.9%)	0 (0%)	3 (1.5%)
Hearing aid use (year)				
Mean (SD)	NA	6.79 (± 6.71)	NA	NA
Missing	NA	15 (7.1%)	NA	NA
Rhyme judgment (% correct)				
Mean (SD)	87.82 (± 11.46)	87.74 (± 11.35)	83.89 (± 14.46)	88.98 (± 10.44)
Rhyme judgment (response time, ms)				
Mean (SD)	1,641 (± 379.7)	1,646 (± 385.3)	1706 (± 358.4)	1,619 (± 378.9)
Weighted WMC (*z*-score)				
Mean (SD)	0.00 (± 0.83)	−0.08 (± 0.80)	−0.42 (± 0.76)	0.20 (± 0.82)

We used the R package ‘MICE’ (van Buuren and Groothuis-Oudshoorn, 2011) to impute missing data in working memory measures. Imputation was performed for the three working memory variables based on the same three variables, in order to keep the imputation results consistent across the group analysis and the hearing aid experience analysis. Because individuals with missing values in all three tests (*n* = 8) had been excluded, the remaining participants had at least one valid score in the three variables. In total, 58 values from 35 participants (13 in the HA group, 11 in the noHA group, and 11 in the NH group) were imputed. The imputation generated five data sets. All following analyses were performed on all five data sets, and the outcomes were generated from pooled results of five analyses.

### Weighted WMC score

We calculated one weighted WMC score for each participant and included it in the models for the following two reasons: (a) we were interested in the concept of working memory as a *latent* factor rather than each working memory measure individually, and (b) the three working memory measures were similar in terms of overall design and test procedure. An Exploratory Factor Analysis (EFA) with a one-factor solution was performed on the three working memory measures for each of the five data sets.

For all five data sets, one factor with eigenvalues greater than 1 was obtained, explaining on average 0.41 of shared variance [KMO ≥ 0.59, Barlett: *x*^2^(3) = 208.6, *p* < 0.001] with the three tests having mean factor loadings of 0.7, 0.72, and 0.46 for reading span, semantic word-pair and VSWM, respectively. We used the factor score estimated based on the factor loading with the Thurstone method (from the R package ‘psych’: Revelle, 2023) as the weighted WMC score for each participant.

### GLMM models

Accuracy and reaction times were analyzed separately in both the group analysis and the hearing aid experience analysis. Generalized linear mixed models (GLMMs), instead of linear mixed models, were used in both analyses, but for different reasons. It was used in the accuracy analyses because we used a binary (correct or incorrect) dependent variable, and it was used in the reaction times analyses because of positively skewed (skew = 1.62) dependent variable. As suggested by Lo and Andrews (2015), a Gamma distribution was used for the reaction time GLMM to avoid a non-normal distribution and heteroscedasticity.

The group analyses were designed to investigate the group difference in the dependence of rhyme judgment performance on WMC. Two models were fitted using the accuracy of all rhyme judgment trials and the reaction time of correct rhyme judgment trials as the dependent variables, respectively. Group, WMC, word-pair type (mismatching or matching), and their interactions were included as fixed effects. Age was also included as a fixed effect. Participant ID and word-pair ID were included as random effects for the intercept.

The hearing-aid experience analyses were designed to investigate the effect of hearing aid experience on rhyme judgment performance in the HA group with the putative moderation of WMC. Two models were fitted using accuracy of all rhyme judgment trials and reaction time of correct rhyme judgment trials as the dependent variables, respectively. Years of hearing aid use, WMC, word-pair type, and their interactions were included as fixed effects. PTA and age were also included as fixed effects to take the severity of hearing loss and aging into account. Participant ID and word-pair ID were included as random effects. In both models, years of hearing aid use was transformed using inverse hyperbolic sine transformation to correct for the right-skewed distribution. Independent variables that were continuous (WMC, age, PTA, and transformed years of hearing aid use) were scaled and centered before entering the model. Distributional assumptions for the within-group errors and random effects in the GLMM were met (Pinheiro and Bates, 2006).

The GLMMs were fitted using the lme4 package (Bates et al., 2015) in R with default ‘bobyqa’ optimizer. For each GLMM, type-III ANOVA (from the package ‘car’: Fox and Weisberg, 2019) was carried out to examine the fixed effects. To better understand the significant interactions, simple effects were examined using the package ‘emmeans’ (Lenth et al., 2023).

## Results

For obvious reasons, the HA group’s average better-ear air-conduction pure tone audiometry at 0.5, 1, 2, and 4 kHz (PTA) was significantly higher than that of the NH group (*t* = 31.11, *p* < 0.001). The noHA group’s average better-ear PTA was significantly higher than that of the NH group (t = −13.49, *p* < 0.001) but significantly lower than that of the HA group (*t* = 6.63, p < 0.001). Moreover, the mean age of the noHA group was higher than both the NH and the HA group (*t* ≤ –5.73, *p* < 0.001), while the NH and HA groups did not differ in age (*t* = −1, *p* = 0.580). The NH group’s weighted WMC score was significantly higher than that of the other two groups (*t* ≥ 3.49, *p* ≤ 0.001), while the HA group had higher weighted WMC than the noHA group (*t* = 2.75, *p* = 0.02).

### Group analyses

To examine the effect of hearing impairment and hearing aid use on the reliance of rhyme judgment performance on WMC, we analyzed the effects of group, WMC, word-pair type, and their interactions on rhyme judgment task performance when controlling for age, both for accuracy and reaction times.

#### Accuracy

WMC and word pair type (matching vs. mismatching) had significant main effects on the accuracy of rhyme judgment [See Table 2: WMC: χ^2^(1) = 45.8, *p* < 0.001; word pair type: χ^2^(1) = 160.53, *p* < 0.001]. The three-way interaction among group, WMC, and word pair type was significant [χ^2^(2) = 8.34, *p* = 0.050].

**Table 2:** Group analyses: ANOVA table for the GLMER on accuracy and reaction time.

	Accuracy			Reaction time		
	$\chi^{2}$	df	*p*	$\chi^{2}$	df	*p*
(Intercept)	102.74	1	<0.001	392,884.37	1	<0.001
Group	0.08	2	0.96	82.52	2	<0.001
WMC	45.8	1	<0.001	3,833.74	1	<0.001
Word pair type	160.53	1	<0.001	11,536.64	1	<0.001
Age	2	1	0.16	42.02	1	<0.001
Group: WMC	4.75	2	0.10	1,259.61	2	<0.001
Group: Word pair type	2.91	2	0.35	612.69	2	<0.001
WMC: Word pair type	1.27	1	0.28	58.73	1	<0.001
Group: WMC: Word pair type	8.34	2	0.05	231.27	2	<0.001

**Figure 1:**
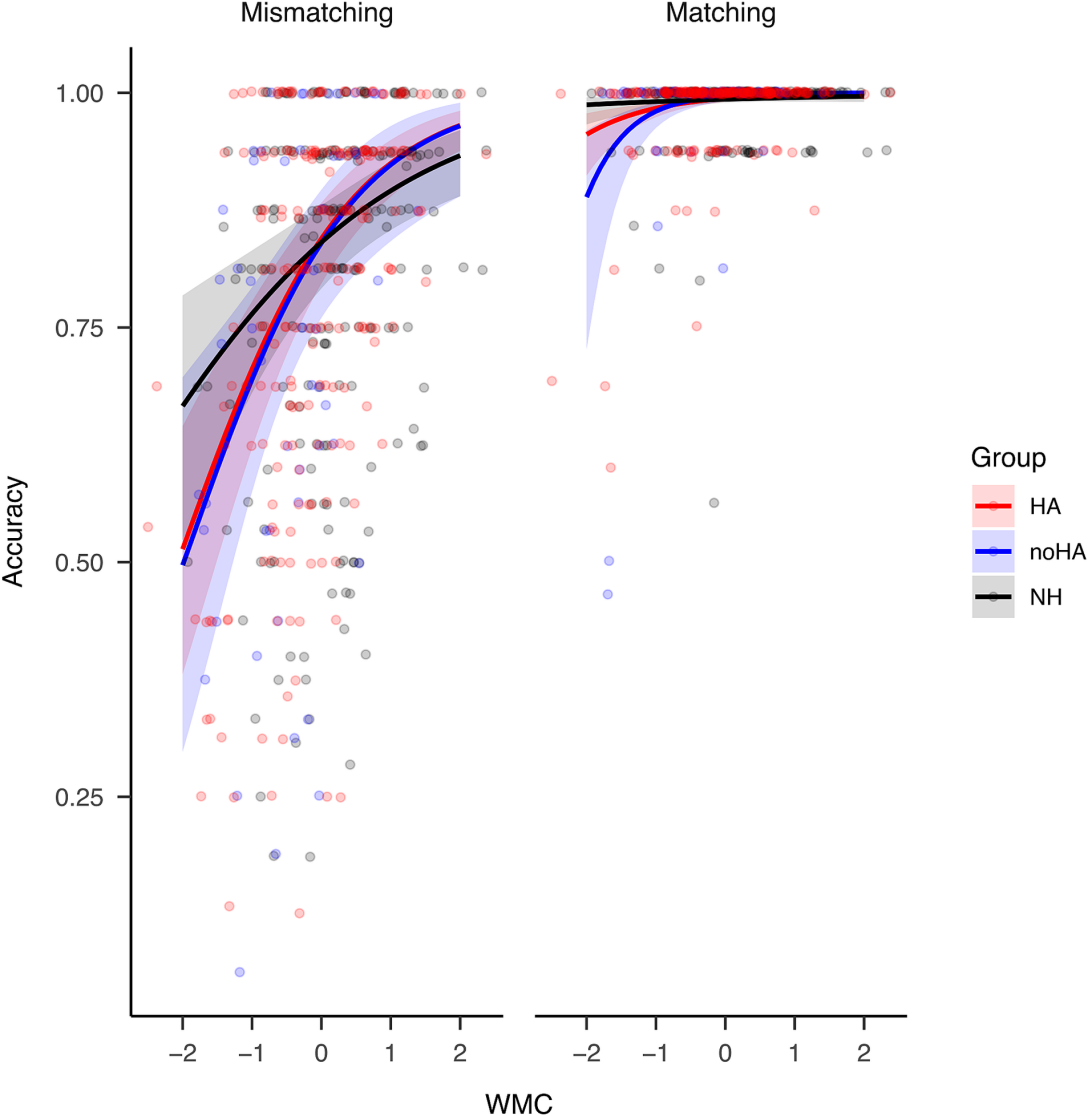
The three-way interaction among group, WMC, and word pair type when predicting rhyme judgment accuracy. Note that the predicted value (*y* axis) has been transformed to reflect accuracy. An increasing WMC led to increasing rhyme judgment accuracy for all groups in mismatching trials, but only for the two groups with hearing impairment in matching trials [HA (red) and noHA (blue)]. The width of the shaded area represents 95% Confidence Interval.

Further analysis on the three-way interaction showed that, when controlling for age, an increasing WMC led to increasing rhyme judgment accuracy for all groups in mismatching trials (Figure 1 left panel; HA: slope = 0.11, *p* < 0.001; noHA: slope = 0.11, *p* < 0.001; NH: slope = 0.07, *p* < 0.001), but only for the two groups with hearing impairment in matching trials (Figure 1 right panel; HA: slope = 0.01, *p* < 0.001; noHA: slope = 0.01, *p* = 0.008; NH: slope = 0, *p* = 0.188). This effect could have been caused by the ceiling effect in the matching trials. No slope difference was found among groups in each trial type. Thus, this pattern shows that WMC was, in general, positively associated with rhyme judgment accuracy, especially for mismatching trials and individuals with hearing impairment. This interpretation, however, should be approached with caution due to the ceiling effect.

#### Reaction time

Group, WMC, word pair type, and age all had significant main effects on the reaction time of rhyme judgment (See Table 2: χ^2^ ≥ 42.02, *p* < 0.001). The three-way interaction among group, WMC, and trial type was significant [χ^2^(2) = 231.27, *p* < 0.001], as were all two-way interactions [χ^2^ ≥ 58.73, *p* < 0.001].

**Figure 2:**
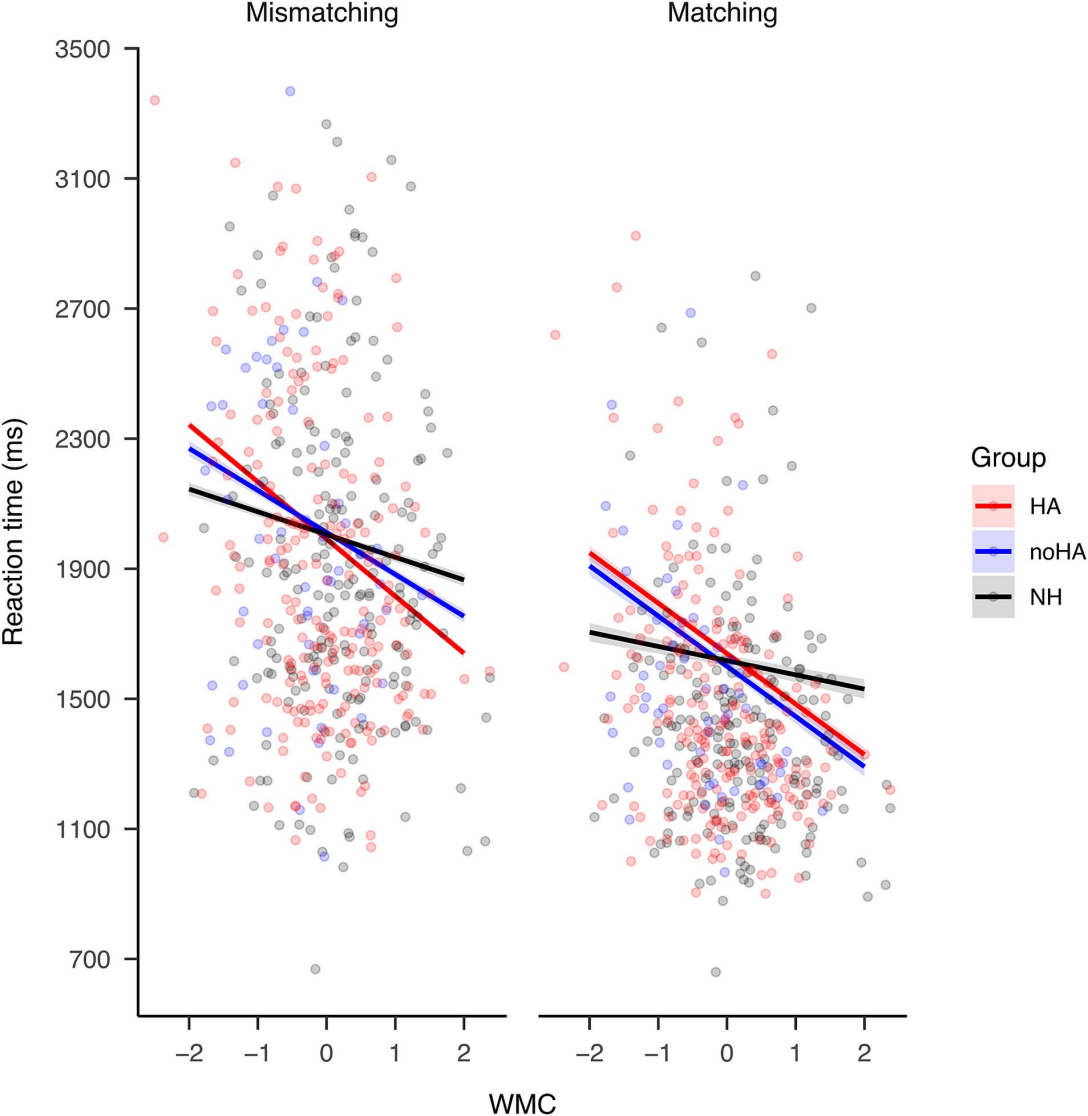
The three-way interaction among group, WMC, and word pair type when predicting rhyme judgment reaction time. An increasing WMC led to faster rhyme judgment reaction time in both trial types for all groups. The rate in which the reaction time decreased was greater in the two groups with hearing impairment [HA (red) and noHA (blue)] than the NH group (black) in both trial types. The rate was even greater in the HA group than the noHA group in mismatching trials. The width of the shaded area represents 95% Confidence Interval.

Further analysis on the three-way interaction among group, WMC, and trial type showed that, when controlling for age, an increasing WMC was associated with faster rhyme judgment reaction time in both trial types for all groups (Figure 2; slope ≤ –43.68, z ≤ –7.34, *p* < 0.001). The rate in which the reaction time decreased was greater in the two groups with hearing impairment (HA and noHA) than the NH group in both trial types (diff ≥ 59.23, *z* ≥ 12.8, *p* < 0.001). The rate was even greater in the HA group than the noHA group in mismatching trials (Figure 2 left panel; diff = −46.65, *z* = −13.32, *p* < 0.001). This pattern shows that WMC was more strongly associated with rhyme judgment speed in individuals with hearing impairment, and that this association was even stronger in hearing aid users than non-hearing aid users in mismatching trials.

### Hearing aid experience analyses

To examine the relationship between hearing aid experience and rhyme judgment with the moderation of working memory, we analyzed the effects of years of hearing aid experience, WMC, word pair type, and their interactions on the rhyme judgment task performance while controlling for hearing level (better-ear PTA) and age in the HA group.

#### Accuracy

In the HA group, years of hearing-aid use, WMC, word pair type, and PTA had significant main effects on the accuracy of rhyme judgment [see Table 3: χ^2^(1) ≥ 5.7, *p ≤* 0.017]. The three-way interaction among years of hearing-aid use, WMC and word pairs type was also significant [χ^2^(1) ≥  6.42, *p ≤* 0.012].

**Table 3:** Hearing aid experience analyses: ANOVA table for the GLMER on accuracy and reaction time.

	Accuracy			Reaction time		
	$\chi^{2}$	df	p	$\chi^{2}$	df	p
(Intercept)	87.52	1	<0.001	101,600.21	1	<0.001
HA years	7.30	1	0.01	0.41	1	0.58
WMC	38.45	1	<0.001	1,261.23	1	<0.001
Word pair type	131.40	1	<0.001	4,761.43	1	<0.001
Age	0.17	1	0.68	7.68	1	0.01
PTA	5.70	1	0.02	59.52	1	<0.001
HA years: WMC	0.52	1	0.47	54	1	<0.001
HA years: Word pair type	0.19	1	0.67	33.4	1	<0.001
WMC: Word pair type	0.93	1	0.36	20.78	1	<0.001
HA years: WMC: Word pair type	6.42	1	0.01	3.01	1	0.12

HA years, years of hearing aid use.

Result showed that when controlling for all other variables, increasing years of hearing-aid use was associated with increasing rhyme judgment accuracy (slope = 0.02, z = 2.53, *p* = 0.011), suggesting that hearing-aid experience was positively associated with rhyme judgment accuracy overall.

**Figure 3:**
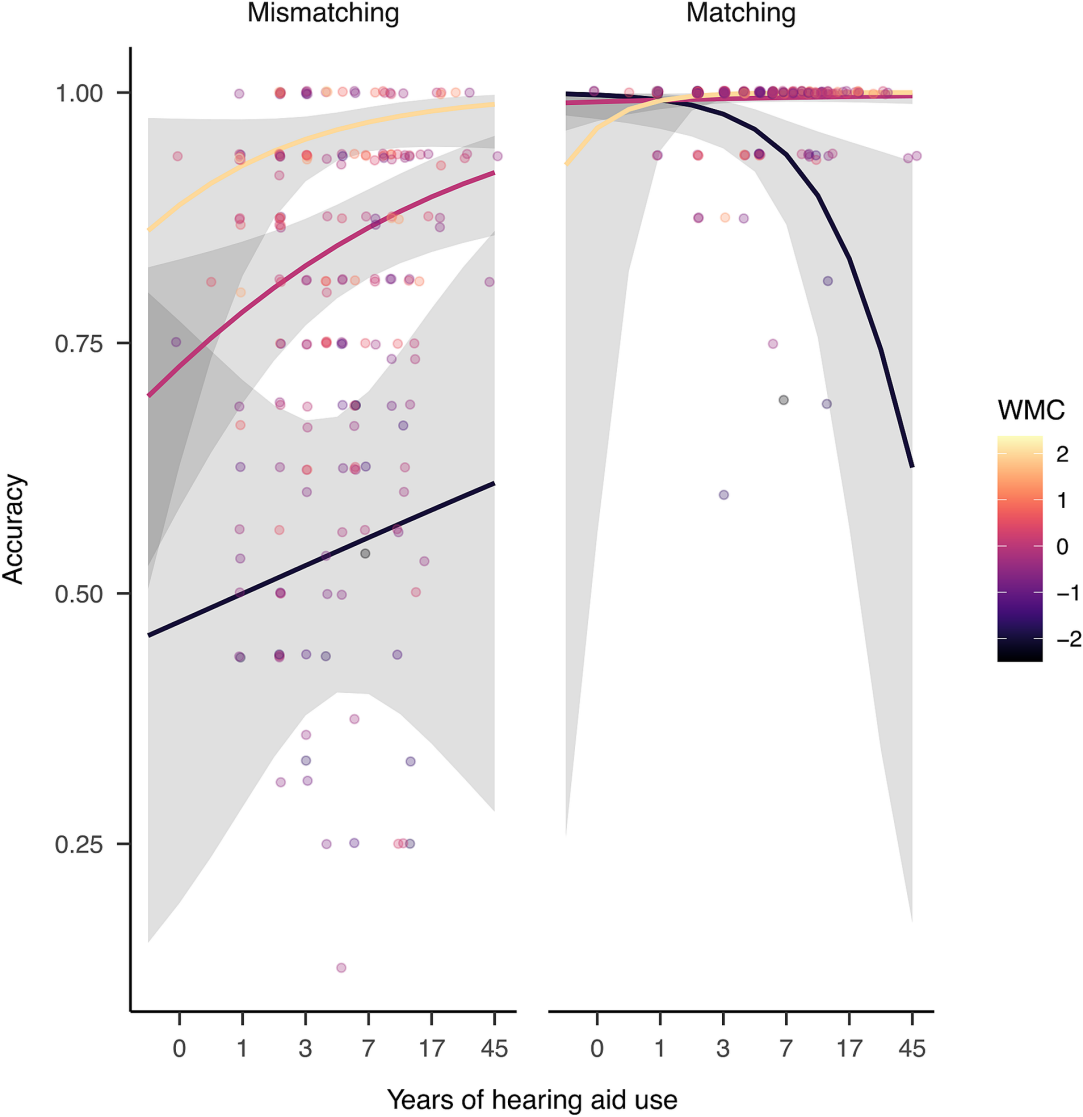
The three-way interaction among years of hearing aid use, WMC, and word pair type when predicting rhyme judgment accuracy. Note that the predicted value (*y* axis) has been transformed to reflect accuracy. In mismatching trials, hearing aid users showed increased accuracy with longer hearing aid use regardless of WMC, albeit only the average WMC individuals reached significance. In matching trials, hearing aid users with below-average WMC showed decreasing accuracy with hearing aid use, while hearing aid users with above-average WMC showed increasing accuracy. The width of the shaded area represents 95% Confidence Interval.

Further analysis following the three-way interaction showed that, when controlling for age and hearing threshold, in mismatching trials, hearing aid users tended to show increased accuracy with longer hearing aid use regardless of WMC, albeit only the average WMC individuals reached significance (Figure 3 left panel; slope = 0.29, z = 2.7, *p* = 0.007). In matching trials, hearing aid users with below-average WMC showed decreasing accuracy with hearing aid use (Figure 3 right panel; hearing aid users with WMC 2 SD below the mean: slope = −1.1, *z* = −2.56, *p* = 0.011), while hearing aid users with above-average WMC showed increasing accuracy (hearing aid users with WMC 2 SD above the mean: slope = 1.51, z = 2.7, *p* = 0.012). This pattern shows that, overall, hearing aid use was positively associated with rhyme judgment accuracy, but for individuals with below-average WMC, the association was negative in matching trials. This interpretation should be approached with caution due to the ceiling effect especially in the matching trials.

#### Reaction time

WMC, word pair type, age, and PTA had significant main effects on the reaction time of rhyme judgment [see Table 3: χ^2^(1) ≥  7.68, *p≤* 0.013]. The interaction between hearing aid use and WMC was significant [χ^2^(1) = 54, *p* < 0.001], as well as for the other two-way interactions [χ^2^(1) ≥ 20.78, *p* < 0.001].

**Figure 4:**
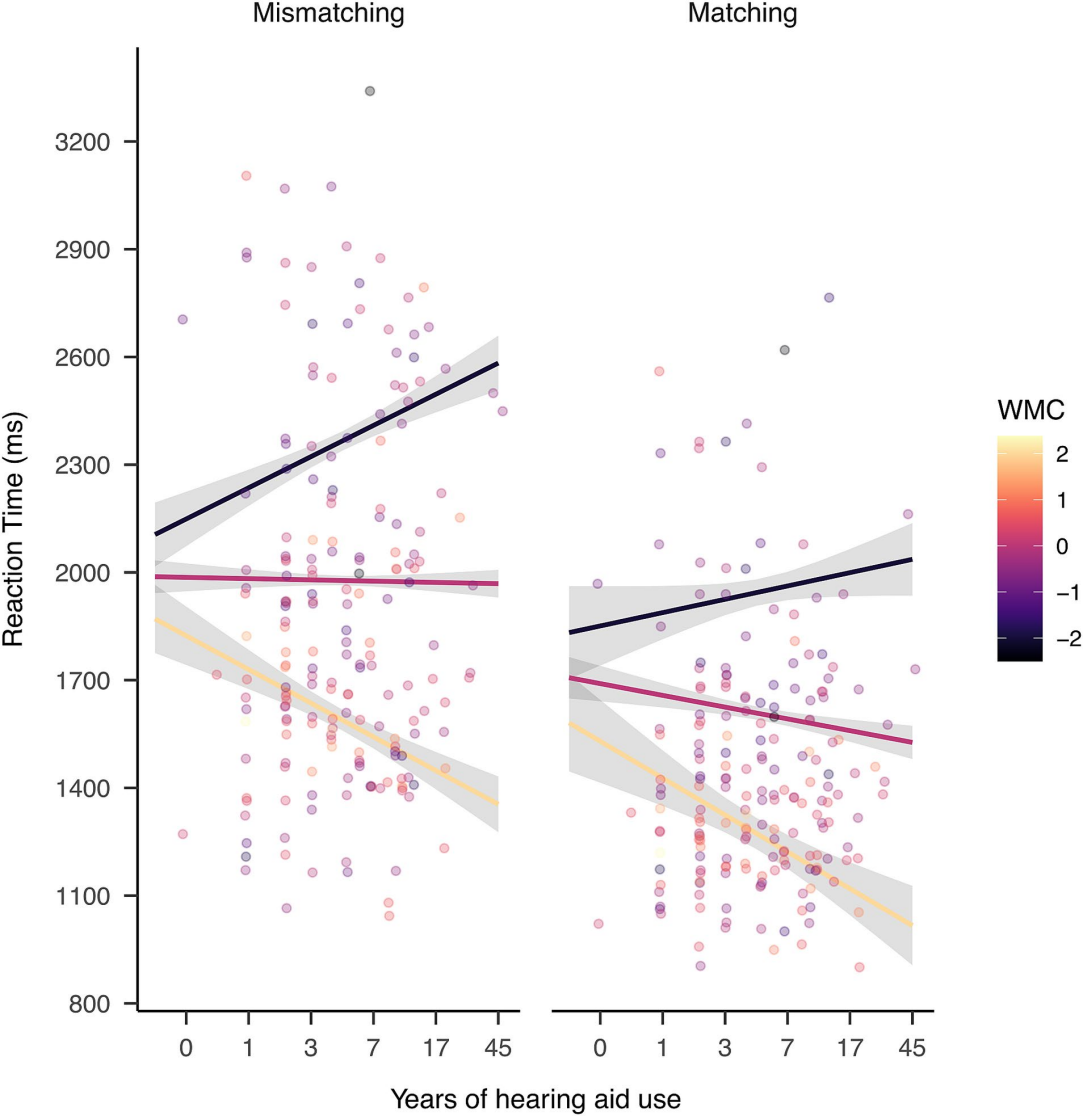
The interaction between WMC and hearing aid use in reaction time. Hearing aid users with above-average WMC (yellow) showed faster rhyme judgment with longer hearing aid use; while those with below-average WMC (black) showed slower rhymer judgment with longer hearing aid use. A similar pattern was observed for both mismatching and matching trials. The width of the shaded area represents 95% Confidence Interval.

Further analysis on the interaction between years of hearing aid use and WMC, and word pair type showed that, when controlling for age and hearing threshold, the way that hearing aid experience and WMC interacted were similar in both word pair types (Figure 4). In both word pair types, increasing years of hearing aid experience was associated with faster rhyme judgment reaction time in participants with above-average WMC (Figure 4; slope ≤ –93.88, *z ≤* –4.89, *p* < 0.001). Longer hearing aid use was associated with slower reaction time in participants with below-average WMC in mismatching word pairs (slope = 86.84, *z* = 6.1, *p* < 0.001) and marginally so in matching word pairs (slope = 37.1, *z* = 1.82, *p* = 0.086). Individuals with average WMC showed faster in reaction time with longer hearing aid use in matching (slope = −32.75, z = −3.97, *p* = 0.005) but not in mismatching word pairs (slope = −3.52, z = −0.57, *p* = 0.582). This pattern shows that working memory moderated the relationship between hearing aid use on rhyme judgment reaction time: above-average WMC was associated with a positive relationship between hearing aid use and rhyme judgment speed, while below-average WMC was associated with a negative relationship.

## Discussion

To investigate how hearing impairment and hearing aid use is associated with phonological processing with the moderating effect of working memory capacity (WMC), we first examined rhyme judgment accuracy and speed while taking WMC into account and controlling for age in the three groups: a group of hearing aid users, a group of hearing-impaired non hearing aid users, and a group of normal hearing controls. We then examined the relationship between years of hearing aid use and rhyme judgment while accounting for WMC and controlling for age and hearing thresholds.

We found that the association between WMC and rhyme judgment performance was stronger in the two groups with hearing impairment regardless of hearing aid use. This is consistent with previous reports (Andersson, 2002; Classon et al., 2013a; Rudner et al., 2019). Moreover, hearing aid use was associated with rhyme judgment performance, interacting with WMC. In the HA group, hearing aid use was not only positively associated with rhyme judgment accuracy, but also interacted with WMC to affect both accuracy and speed. Specifically, better WMC was associated with stronger positive relationship between hearing aid use and rhyme judgment: participants with average and above-average WMC showed better rhyme judgement performance with increased hearing aid use, while participants with lower WMC did not. These findings, though correlational in nature, may suggest that hearing aid use potentially leads to improvements on visual rhyme judgment, a non-auditory task that reflects phonological processing. Additionally, these findings also highlight the role of working memory as a moderator in shaping the effect of hearing aid use.

### The effect of hearing impairment on phonological processing

The current finding suggests that individuals with hearing impairment rely more on working memory for phonological processing tasks (cf. Classon et al., 2013a; Classon et al., 2013b), as evidenced by the greater positive effect of WMC in the HA and noHA groups compared to the NH group. With the group that were hearing impaired but did not use hearing aids (noHA), we were able to rule out the possibility that hearing aid use alone contributed to the increase in the involvement of WMC in rhyme judgment. This finding aligned with the proposal that hearing impairment led to deterioration in phonological processing and that individuals with hearing impairment compensate for this deterioration by employing strategies that heavily rely on working memory (Classon et al., 2013a; Andersson, 2002). Curiously, in the current study, the groups with hearing impairment showed no disadvantage in rhyme judgment accuracy or reaction time compared to the NH group when WMC and age was controlled at average. This was different from previous studies that reported lower accuracy and longer reaction time in individuals with hearing impairment (Andersson and Lyxell, 1999; Andersson, 2002; Lyxell et al., 1994, 1998). It was possible that age and WMC contributed to the differences found in previous studies. In addition, and perhaps the most likely explanation, is that those studies employed participants with higher degrees of hearing impairment, which in turn affected phonological representations to a larger extent (Rönnberg et al., 2013).

### The effect of hearing aid use on phonological processing

We found that hearing aid use was positively associated with rhyme judgment performance, controlling for age and hearing level. Years of hearing aid use had a positive main effect on accuracy. Furthermore, hearing aid use also was associated with rhyme judgment speed, albeit moderated by WMC: for hearing aid users with average and above-average WMC, hearing aid use had a positive assoication with speed, while for hearing aid users with below-average WMC, the association was negative. Though correlational in nature, these findings may be interpreted to suggest that longer use of hearing aids lead to better performance in phonological processing.

The current study, to the best of our knowledge, is the first to link hearing aid use with better phonological processing. This finding could have clinical relevance and potential implications for the hearing aid industry. In a previous study that solely examined hearing aid users (Rudner et al., 2019), no effect of hearing aid use on rhyme judgment performance was found. This difference could be caused by the different statistical model structure between studies. For example, Rudner et al. included lexical access speed as an independent variable to examine the underlying mechanism of phonological processing, while in the present study the focus was on the interaction between hearing aid use and WMC. The interaction was found to be significant, as WMC was shown to greatly influence how hearing aid use predicts rhyme judgment reaction time. Provided that, it remains to be seen whether lexical access speed was what was facilitated by hearing aid use in a phonological processing task.

It may appear surprising that there was a negative relationship between hearing aid use and rhyme judgment speed in individuals with below-average WMC. Because we have controlled for hearing thresholds and age, it was unlikely that worse hearing and older age—factors likely associated with slower speed—was contributing to this effect. One factor that may have contributed to this effect was the duration of hearing impairment, which has been shown to correlate with phonological skill deterioration in deafened individuals (Classon et al., 2013a; Lyxell et al., 1994, 1998). The duration of impairment was not controlled because we encountered challenges in verifying the reliability of related data at the time of analysis. As a result, long hearing aid use in the current study was often accompanied by long hearing impairment, and the negative effect of long hearing impairment might outweigh the positive effect of long hearing aid use, leading to the net worsening in rhyme judgment speed in some participants. If this is the case, then the rhyme judgment speed in those participants should be faster than if they had not used hearing aid. Future studies that compare the rhyme judgment speed between hearing aid users and non-hearing aid users with hearing impairment controlling for the duration of hearing impairment should be able to provide evidence for this argument. Furthermore, the duration of hearing impairment should have also affected hearing aid users with average and above-average WMC, which indicated that for them, the benefit they obtained from hearing aid use outweighed the worsening caused by longer hearing impairment. In sum, the current result pattern leads to the speculations that WMC protects against the negative effects of hearing impairment when technical intervention such as hearing aid is implemented (cf. Ng and Rönnberg, 2020).

WMC seemed to be an important moderator as to the effect of hearing aid use on phonological processing speed. One possibility is that WMC affects how effectively hearing aid users utilize the signal processing capabilities of their hearing aids: those users with above-average WMC may utilize hearing aid signal processing to a fuller extent which benefited their updating of phonological representation, especially with more advanced signal processing in the hearing aid (Lunner et al., 2009; Rönnberg et al., 2022). Thus, WMC would compensate for the worsening caused by longer hearing impairment, showing faster speed in phonological processing tasks with better WMC. The benefit of WMC could not outweigh the worsening caused by longer hearing impairment, showing slower rhyme judgment (but still faster than if they had not used hearing aids).

Nevertheless, the slower speed in rhyme judgment may not necessarily be unfavorable for hearing aid users with below-average WMC. There has been evidence that, when phonological representation weakens, as is the case with individuals with hearing impairment, some individuals may resort to a lexical-semantic strategy in reading or rhyme judgment task performance, bypassing the ‘normal’ route that accesses phonological information (Classon et al., 2013b; Lazard et al., 2010). The lexical strategy, albeit faster and less WMC-dependent, is presumably accompanied by a series of functional reorganizations of the brain which in, e.g., CI candidates have been found to predict poor speech perception performance after CI (Lazard et al., 2011). Thus, we speculate that the slowing of rhyme judgment speed with longer hearing aid use in those with below-average WMC indicates that they may have kept using the slower and WMC-dependent phonological strategy, avoiding the functional reorganizations that have a negative impact on speech perception in hearing rehabilitation. Additional neuroimaging studies involving the population are needed to provide stronger evidence supporting this possibility.

### The dependence of phonological processing on WMC

Interpreting the current results from a different perspective, hearing aid use seemed to increase reliance on WMC in rhyme judgment performance. This was seen in the stronger association with WMC in the HA than the noHA group in mismatching trials (Figure 2) and the larger impact of WMC on longer hearing aid users (Figure 4). This finding contradicts a previous study that also reported an interaction between WMC and hearing aid use. Ng and Rönnberg (2020) found that reliance on WMC was stronger for relatively less experienced (2–5 years) hearing aid users compared to more experienced (5+ years) users in a speech recognition task in stationary noise. The key difference between this and the current finding may have been caused by the different nature of the tasks. In the speech perception in noise task, the enhanced involvement of working memory signals less automatic and more effortful processing (Ng and Rönnberg, 2020; Rönnberg et al., 2013). However, in visual rhyme judgment, the enhanced involvement of working memory may also show a different strategy. As in the previous section, the increase in dependence on WMC for prolonged use of hearing aids may not be unfavorable, because it may hint at the preservation of the phonological strategy, thereby indicating the avoidance of the functional organizations of the brain related to poor speech perception outcomes. This contrast suggests that the involvement of WMC in task performance differs according to the characteristics of the task and requires careful interpretation.

Moreover, the current study showed that hearing impairment was associated with increased WMC dependency in phonological processing, and hearing aid use seemed to strengthen this dependency even further. This finding may suggest that hearing aid use did not simply revert the influence of hearing impairment, but led users with different WMC to adapt to the changes in environment in different ways with an overall positive effect.

### Mismatching and matching trials in rhyme judgment

In the rhyme judgment task, whether orthographic information matched with rhyme or not affected performance accuracy and speed together with hearing impairment, hearing aid use, and WMC. To begin with, the matching trials showed no change with WMC in NH participants but revealed a positive effect of WMC in groups with hearing impairment, while in mismatching trials all groups showed a positive effect (Figure 1), driving the significant three-way interaction in the group analysis.

Regarding hearing aid use, its interaction with WMC had a larger impact on rhyme judgment speed for mismatching than matching trials. This is shown in the difference in WMC-reliance between the noHA group and HA group in mismatching but not matching trials (Figure 2). The interaction had a greater impact on accuracy for matching than mismatching trials, as seen in the different influence hearing aid use had on accuracy depending on WMC in matching but not mismatching trials (Figure 3). In other words, in mismatching trials, speed was sensitive to differences in hearing aid use and WMC, while in matching trials, accuracy was sensitive to differences in hearing aid use and WMC.

In the literature, mismatching trials were thought to require more phonological processing than matching trials. However, Rudner et al. (2019) argued that both trial types required phonological processing because when they were presented randomly, the participant would need to access phonological information for all trials no matter the orthographical similarity to reach optimal performance. In their research, Rudner et al. (2019) found that rhyme judgment speed in matching but not mismatching trials was predicted by working memory over and beyond lexical access speed. The current study did not find a similar outcome, likely because lexical access speed was not included in the model.

## Limitations and future directions

One limitation of the current study is its cross-sectional and observational nature, which makes it difficult to establish causal relationships. For example, we interpreted the interaction between WMC and hearing aid use to suggest that individuals with above-average WMC benefit more from hearing aid use in terms of phonological processing, but the interaction may also be caused by individuals with better phonological processing and above-average WMC being more likely to use hearing aid for longer. Future studies could employ longitudinal designs to explore how the relationship between hearing aid use, WMC, and phonological processing evolves over time. These studies could determine whether the cognitive benefits of hearing aid use accumulate with prolonged use and whether these benefits vary based on the severity and duration of hearing impairment, potentially providing causal evidence for the benefit of hearing aid use.

Another potential limitation is that the groups differed significantly in their age, hearing levels (especially between the noHA and HA groups), and WMC scores. While the difference in age was controlled by including it as a covariate in the model, the degree of hearing loss might still affect the outcomes of the group analysis. However, our key comparison between the noHA and NH groups showed that the noHA group, even with relatively mild hearing loss, exhibited a greater reliance on WMC. This finding underscores the robustness of the observed effects despite these differences. The variation in WMC scores across groups also raises concerns. The observed group-by-WMC interaction in predicting rhyme judgment performance could partly reflect underlying group differences in mean WMC rather than a purely interaction-driven relationship. Although the inclusion of WMC as a covariate in the model helps control for these differences statistically, it does not fully eliminate the potential influence of baseline differences. Future studies with larger sample sizes could address this limitation by matching participants across groups on age, degree of hearing loss, and WMC to minimize baseline differences.

Previous studies showed that the processing of a R + O–trial is likely to differ from that of a R + O–trial, even though they are both mismatching trials, and the same is true for the two types of matching trials (e.g., Andersson, 2002). In this study, the small number of trials in each category precluded analyses in this aspect. Thus, future research investigating the effect of hearing aid use and WMC on each category of rhyme judgment trials would refine our understanding of how these factors interact to influence psycholinguistic abilities including phonological processing.

Future research could also explore the underlying mechanisms that facilitate the interaction between WMC and hearing aid use in phonological processing. As previously discussed, the current findings could be interpreted to suggest that WMC affects how effectively hearing aid users utilize the signal processing capabilities of their devices, or that hearing aid use increases reliance on WMC in phonological performance, reflecting a shift in cognitive strategy. Future neuroimaging studies could investigate these possibilities by examining brain activity patterns associated with rhyme judgment performance. Such studies would help clarify whether WMC enables more efficient use of hearing aid signal processing or whether hearing aid use fosters compensatory cognitive strategies.

Understanding the interaction between WMC and hearing aid use has practical implications for designing interventions to promote phonological skills in hearing aid users. These skills are vital for language processing across modalities. Tailored rehabilitation programs could focus on optimizing hearing aid settings to support phonological processing. For example, users with lower WMC might benefit from simplified signal processing.

## Conclusion

In conclusion, this study contributes to the understanding of the intricate relationship among hearing impairment, hearing aid use, and cognitive functions (especially working memory capacity, WMC) in influencing phonological processing—a linguistic ability beyond the auditory domain. Our findings suggest that hearing impairment exacerbates the reliance on WMC for phonological processing tasks (cf. the ELU model, 2013, 2022). This suggests that cognitive compensatory mechanisms may be at play to mitigate the challenges posed by auditory distortions and deprivations. Notably, the study also sheds light on the role of hearing aids in this context. Hearing aid use might generally facilitate phonological processing performance, with the effectiveness moderated by the user’s WMC. This underscores the necessity of considering cognitive factors when evaluating the benefits of hearing aids, as individuals with lower WMC may not experience the same level of improvement as those with higher WMC.

## Data Availability

The data analyzed in this study is subject to the following licenses/restrictions: The n200 data base can be accessed by requesting variables from Rönnberg (PI) after having signed a Data Transfer Agreement. Requests to access these datasets should be directed to Jerker Rönnberg, jerker.ronnberg@liu.se.

## References

[ref1] Amieva H. Ouvrard C. Giulioli C. Meillon C. Rullier L. Dartigues J.-F. (2015). Self-reported hearing loss, hearing aids, and cognitive decline in elderly adults: a 25-year study. J. Am. Geriatr. Soc. 63, 2099–2104. doi: 10.1111/jgs.13649, PMID: 26480972

[ref2] Andersson U. (2002). Deterioration of the phonological processing skills in adults with an acquired severe hearing loss. Eur. J. Cogn. Psychol. 14, 335–352. doi: 10.1080/09541440143000096

[ref3] Andersson U. Lyxell B. (1999). Phonological deterioration in adults with an acquired severe hearing impairment. A deterioration in long-term memory or working memory? Scand. Audiol. 28, 241–247. doi: 10.1080/010503999424671, PMID: 10572969

[ref4] Baddeley A. (1992). Working memory. Science 255, 556–559. doi: 10.1126/science.1736359, PMID: 1736359

[ref5] Baddeley A. Thomson N. Buchanan M. (1975). Word length and the structure of short-term memory. J. Verbal Learn. Verbal Behav. 14, 575–589. doi: 10.1016/S0022-5371(75)80045-4

[ref6] Bates D. Mächler M. Bolker B. Walker S. (2015). Fitting linear mixed-effects models using lme4. J. Stat. Softw. 67, 1–48. doi: 10.18637/jss.v067.i01, PMID: 27620283

[ref7] Booth J. R. Burman D. D. Meyer J. R. Gitelman D. R. Parrish T. B. Mesulam M. M. (2004). Development of brain mechanisms for processing orthographic and phonologic representations. J. Cogn. Neurosci. 16, 1234–1249. doi: 10.1162/0898929041920496, PMID: 15453976 PMC1382289

[ref8] Bucholc M. Bauermeister S. Kaur D. McClean P. L. Todd S. (2022). The impact of hearing impairment and hearing aid use on progression to mild cognitive impairment in cognitively healthy adults: an observational cohort study. Alzheimers Dementia 8:e12248. doi: 10.1002/trc2.12248, PMID: 35229022 PMC8863441

[ref9] Classon E. Rudner M. Johansson M. Rönnberg J. (2013b). Early ERP signature of hearing impairment in visual rhyme judgment. Front. Psychol. 4:241. doi: 10.3389/fpsyg.2013.00241, PMID: 23653613 PMC3644703

[ref10] Classon E. Rudner M. Rönnberg J. (2013a). Working memory compensates for hearing related phonological processing deficit. J. Commun. Disord. 46, 17–29. doi: 10.1016/j.jcomdis.2012.10.001, PMID: 23157731

[ref11] Daneman M. Carpenter P. A. (1980). Individual differences in working memory and reading. J. Verbal Learn. Verbal Behav. 19, 450–466. doi: 10.1016/S0022-5371(80)90312-6

[ref12] Dawes P. Emsley R. Cruickshanks K. J. Moore D. R. Fortnum H. Edmondson-Jones M.. (2015). Hearing loss and cognition: the role of hearing aids. Soc. Isol. Depression 10:e0119616. doi: 10.1371/journal.pone.0119616, PMID: 25760329 PMC4356542

[ref13] Fox J. Weisberg S. (2019). An R companion to applied regression. Third edition. Sage, Thousand Oaks CA. Available at: https://www.john-fox.ca/Companion/

[ref14] Goswami U. (2012). “Phonological Representation” in Encyclopedia of the sciences of learning. ed. Seel N. M. (New York, NY: Springer US), 2625–2627.

[ref15] Homman L. Danielsson H. Rönnberg J. (2023). A structural equation mediation model captures the predictions amongst the parameters of the ease of language understanding model. Front. Psychol. 14, 1–16. doi: 10.3389/fpsyg.2023.1015227, PMID: 36936006 PMC10020708

[ref16] Lazard D. S. Giraud A.-L. (2017). Faster phonological processing and right occipito-temporal coupling in deaf adults signal poor cochlear implant outcome. Nat. Commun. 8:1. doi: 10.1038/ncomms14872, PMID: 28348400 PMC5379061

[ref17] Lazard D. S. Giraud A. L. Truy E. Lee H. J. (2011). Evolution of non-speech sound memory in postlingual deafness: implications for cochlear implant rehabilitation. Neuropsychologia 49, 2475–2482. doi: 10.1016/j.neuropsychologia.2011.04.025, PMID: 21557954

[ref18] Lazard D. S. Lee H. J. Gaebler M. Kell C. A. Truy E. Giraud A. L. (2010). Phonological processing in post-lingual deafness and cochlear implant outcome. NeuroImage 49, 3443–3451. doi: 10.1016/j.neuroimage.2009.11.013, PMID: 19931402

[ref19] Lenth R. V. Bolker B. Buerkner P. Giné-Vázquez I. Herve M. Jung M.. (2023). Emmeans: Estimated marginal means, aka least-squares means (1.8.9) [computer software]. https://cran.r-project.org/web/packages/emmeans/index.html (Accessed November 16, 2023).

[ref20] Lin F. R. Pike J. R. Albert M. S. Arnold M. Burgard S. Chisolm T.. (2023). Hearing intervention versus health education control to reduce cognitive decline in older adults with hearing loss in the USA (ACHIEVE): a multicentre, randomised controlled trial. Lancet 402, 786–797. doi: 10.1016/S0140-6736(23)01406-X, PMID: 37478886 PMC10529382

[ref21] Lin F. R. Yaffe K. Xia J. Xue Q.-L. Harris T. B. Purchase-Helzner E.. (2013). Hearing loss and cognitive decline in older adults. JAMA Intern. Med. 173, 293–299. doi: 10.1001/jamainternmed.2013.1868, PMID: 23337978 PMC3869227

[ref22] Livingston G. Sommerlad A. Orgeta V. Costafreda S. G. Huntley J. Ames D.. (2017). Dementia prevention, intervention, and care. Lancet 390, 2673–2734. doi: 10.1016/S0140-6736(17)31363-6, PMID: 28735855

[ref23] Lo S. Andrews S. (2015). To transform or not to transform: using generalized linear mixed models to analyse reaction time data. Front. Psychol. 6:1171. doi: 10.3389/fpsyg.2015.01171, PMID: 26300841 PMC4528092

[ref24] Lunner T. Rudner M. Rönnberg J. (2009). Cognition and hearing aids. Scand. J. Psychol. 50, 395–403. doi: 10.1111/j.1467-9450.2009.00742.x, PMID: 19778387

[ref25] Lyxell B. Andersson J. Andersson U. Arlinger S. Bredberg G. Harder H. (1998). Phonological representation and speech understanding with cochlear implants in deafened adults. Scand. J. Psychol. 39, 175–179. doi: 10.1111/1467-9450.393075, PMID: 9800533

[ref26] Lyxell B. Andersson U. Borg E. Ohlsson I.-S. (2003). Working-memory capacity and phonological processing in deafened adults and individuals with a severe hearing impairment. Int. J. Audiol. 42, 86–89. doi: 10.3109/14992020309074628, PMID: 12918614

[ref27] Lyxell B. Rönnberg J. Samuelsson S. (1994). Internal speech functioning and speechreading in deafened and Normal hearing adults. Scand. Audiol. 23, 179–185. doi: 10.3109/01050399409047505, PMID: 7997835

[ref28] MacSweeney M. Goswami U. Neville H. (2013). The neurobiology of rhyme judgment by deaf and hearing adults: an ERP study. J. Cogn. Neurosci. 25, 1037–1048. doi: 10.1162/jocn_a_00373, PMID: 23448521 PMC4872821

[ref29] Maharani A. Pendleton N. Leroi I. (2019). Hearing impairment, loneliness, social isolation, and cognitive function: longitudinal analysis using English longitudinal study on ageing. Am. J. Geriatr. Psychiatry 27, 1348–1356. doi: 10.1016/j.jagp.2019.07.010, PMID: 31402088

[ref30] Maki W. S. (2007). Judgments of associative memory. Cogn. Psychol. 54, 319–353. doi: 10.1016/j.cogpsych.2006.08.002, PMID: 16982044

[ref31] Ng E. H. N. Rönnberg J. (2020). Hearing aid experience and background noise affect the robust relationship between working memory and speech recognition in noise. Int. J. Audiol. 59, 208–218. doi: 10.1080/14992027.2019.1677951, PMID: 31809220

[ref32] Oakhill J. Kyle F. (2000). The relation between phonological awareness and working memory. J. Exp. Child Psychol. 75, 152–164. doi: 10.1006/jecp.1999.2529, PMID: 10620378

[ref33] Pillay S. B. Stengel B. C. Humphries C. Book D. S. Binder J. R. (2014). Cerebral localization of impaired phonological retrieval during rhyme judgment. Ann. Neurol. 76, 738–746. doi: 10.1002/ana.24266, PMID: 25164766 PMC4214892

[ref34] Pinheiro J. Bates D. (2006). Mixed-effects models in S and S-PLUS. New York, NY: Springer Science & Business Media.

[ref35] Revelle W. (2023). Psych: Procedures for psychological, psychometric, and personality research (2.3.9) [computer software]. Available at: https://cran.r-project.org/web/packages/psych/index.html (Accessed November 16, 2023).

[ref36] Rönnberg J. Arlinger S. Lyxell B. Kinnefors C. (1989). Visual evoked potentials. J. Speech Lang. Hear. Res. 32, 725–735. doi: 10.1044/jshr.3204.725 2601304

[ref37] Rönnberg J. Danielsson H. Rudner M. Arlinger S. Sternäng O. Wahlin Å.. (2011). Hearing loss is negatively related to episodic and semantic long-term memory but not to short-term memory. J. Speech Lang. Hear. Res. 54, 705–726. doi: 10.1044/1092-4388(2010/09-0088), PMID: 20884779

[ref38] Rönnberg J. Hygge S. Keidser G. Rudner M. (2014). The effect of functional hearing loss and age on long-and short-term visuospatial memory: evidence from the UK biobank resource. Front. Ageing Neurosci. 6:326. doi: 10.3389/fnagi.2014.00326, PMID: 25538617 PMC4260513

[ref39] Rönnberg J. Lunner T. Ng E. H. N. Lidestam B. Zekveld A. A. Sörqvist P.. (2016). Hearing impairment, cognition and speech understanding: exploratory factor analyses of a comprehensive test battery for a group of hearing aid users, the n200 study. Int. J. Audiol. 55, 623–642. doi: 10.1080/14992027.2016.1219775, PMID: 27589015 PMC5044772

[ref40] Rönnberg J. Lunner T. Zekveld A. Sörqvist P. Danielsson H. Lyxell B.. (2013). The ease of language understanding (ELU) model: theoretical, empirical, and clinical advances. Front. Syst. Neurosci. 7:31. doi: 10.3389/fnsys.2013.00031, PMID: 23874273 PMC3710434

[ref41] Rönnberg J. Signoret C. Andin J. Holmer E. (2022). The cognitive hearing science perspective on perceiving, understanding, and remembering language: the ELU model. Front. Psychol. 13:967260. doi: 10.3389/fpsyg.2022.967260, PMID: 36118435 PMC9477118

[ref42] Rudner M. Danielsson H. Lyxell B. Lunner T. Rönnberg J. (2019). Visual rhyme judgment in adults with mild-to-severe hearing loss. Front. Psychol. 10:1149. doi: 10.3389/fpsyg.2019.01149, PMID: 31191388 PMC6546845

[ref43] Rugg M. D. Barrett S. E. (1987). Event-related potentials and the interaction between orthographic and phonological information in a rhyme-judgment task. Brain Lang. 32, 336–361. doi: 10.1016/0093-934X(87)90132-5, PMID: 3690257

[ref44] Sanders M. E. Kant E. Smit A. L. Stegeman I. (2021). The effect of hearing aids on cognitive function: a systematic review. PLoS One 16:e0261207. doi: 10.1371/journal.pone.0261207, PMID: 34972121 PMC8719768

[ref45] van Buuren S. Groothuis-Oudshoorn K. (2011). Mice: multivariate imputation by chained equations in R. J. Stat. Softw. 45, 1–67. doi: 10.18637/jss.v045.i03

